# Transcranial alternating current stimulation in affecting cognitive impairment in psychiatric disorders: a review

**DOI:** 10.1007/s00406-023-01687-7

**Published:** 2023-09-08

**Authors:** Nina Biačková, Andrea Adamová, Monika Klírová

**Affiliations:** 1 Neurostimulation Department, National Institute of Mental Health, Klecany, Czech Republic; 2 Third Faculty of Medicine, Charles University, Prague, Czech Republic

**Keywords:** Transcranial alternating current stimulation (tACS), Non-invasive brain stimulation (NIBS), Cognitive impairment, Psychiatry, Dementia, Schizophrenia, Depression, OCD, ADHD, SUD, COVID-19

## Abstract

Transcranial alternating current stimulation (tACS) is a non-invasive brain stimulation method that, through its manipulation of endogenous oscillations, can affect cognition in healthy adults. Given the fact that both endogenous oscillations and cognition are impaired in various psychiatric diagnoses, tACS might represent a suitable intervention. We conducted a search of Pubmed and Web of Science databases and reviewed 27 studies where tACS is used in psychiatric diagnoses and cognition change is evaluated. TACS is a safe and well-tolerated intervention method, suitable for multiple-sessions protocols. It can be administered at home, individualized according to the patient'’s anatomical and functional characteristics, or used as a marker of disease progression. The results are varying across diagnoses and applied protocols, with some protocols showing a long-term effect. However, the overall number of studies is small with a great variety of diagnoses and tACS parameters, such as electrode montage or used frequency. Precise mechanisms of tACS interaction with pathophysiological processes are only partially described and need further research. Currently, tACS seems to be a feasible method to alleviate cognitive impairment in psychiatric patients; however, a more robust confirmation of efficacy of potential protocols is needed to introduce it into clinical practise.

## Introduction

Transcranial alternating current stimulation (tACS), which works by applying alternating current of low intensity through the scalp to the brain, is one of the so-called non-invasive brain stimulation methods (NIBS). The current intensity is too small to induce action potential by itself, however, it causes rhythmic changes of membrane potential, i.e., shifting the neuronal populations closer to hyperpolarisation or depolarisation, and thereby influences the spike timing [1]. By applying electrical current at a certain frequency, it interacts with naturally occurring endogenous oscillations (EOs). The exact mechanism of this interaction depends on the used current intensity and delivered dose. Five types of local interaction are described: stochastic resonance, rhythm resonance, temporal biasing of neuronal spikes, entrainment of network patterns, and imposed patterns (for full review see [2]). The effect of tACS can be observed during stimulation, so-called online-effect, supposedly through entrainment of EOs. However, a long-term (offline) effect can also be induced by influencing synaptic plasticity [1, 3]. Current reviews describe effects of tACS on various aspects of psychiatric disorders [4]; however, to the authors’ knowledge, the effect of tACS on cognition across the disorders has not yet been fully assessed. Some form of cognitive impairment (CI) is present in various psychiatric diagnoses, including Alzheimer’s dementia (AD), schizophrenia (SCH), major depressive disorder (MDD), obsessive–compulsive disorder (OCD), attention deficit and hyperactivity disorder (ADHD), substance use disorder (SUD), and post-acute sequelae of COVID-19 (PASC). EOs are associated with cognitive functions on a specific [5–9] or general level [10] and therefore may be connected to these impairments. Manipulation with EOs by tACS results in changes in cognitive performance in healthy adults (for full review see [11], for meta-analysis of effects see [12]). In studies with healthy participants, tACS is used to reveal the precise mechanisms underlying the particular cognitive domains, such as modulating conflict and error processing through frontal midline theta-tACS [13], but also for neuroenhancement, such as that of working memory [14] or long-term memory [15]. Therefore, tACS might constitute a suitable intervention targeting CI in psychiatric patients. This review evaluates studies attempting such intervention and connects the results to described pathological findings.

## Methods

**Fig. 1:**
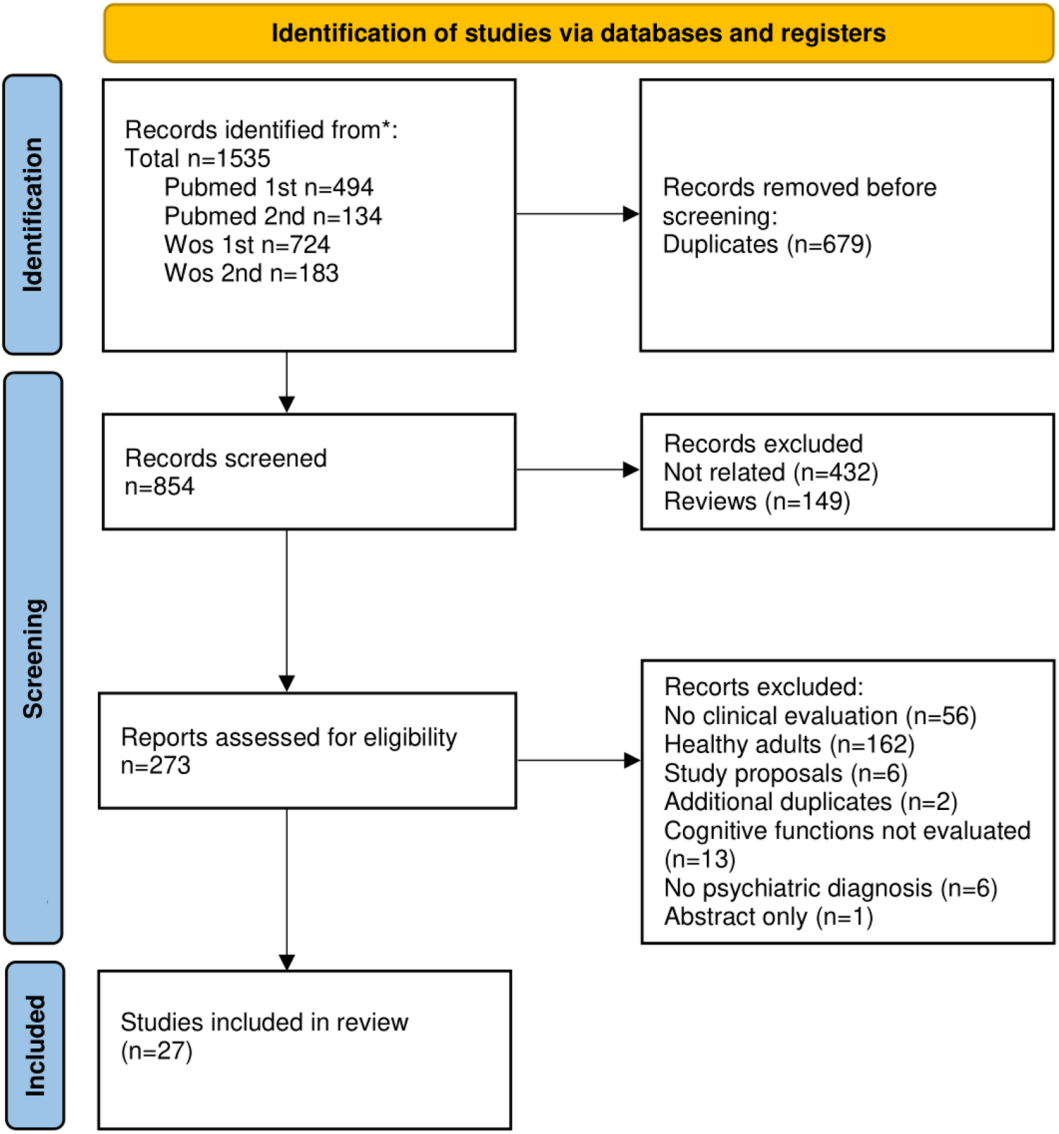
PRISMA diagram detailing the selection of the papers included in this review

The studies in this review were filtered according to the PRISMA 2020 diagram [16]. The search was conducted on 24 March 2023 using two databases: Pubmed and Web of Science. Two groups of terms were used separately: “(("tacs") OR ("transcranial alternating current stimulation") OR ("alternating current stimulation")) AND (("cognitive function") OR ("cognitive impairment") OR ("cognition") OR (cognit*) OR (memory) OR (attention))”, and *“(("tacs") OR ("transcranial alternating current stimulation") OR ("alternating current stimulation")) AND ((OCD) OR (ADHD) OR (schizophrenia) OR (depression) OR (anxiety) OR (dementia))”*, creating four separate sets of results. After automatic filtering of duplicates, n = 854 records remained for screening. Through the screening of title and/or abstract, n = 432 records were identified as unrelated to the topic, and n = 149 records were identified as reviews and therefore discarded from this review. Full-text versions of the remaining n = 273 records were further assessed to determine their eligibility. After exclusion of records without any clinical evaluation (n = 56), studies involving healthy adults (n = 162), study proposals (n = 6), duplicate records (n = 2), studies involving non-psychiatric diagnoses (n = 6), studies without evaluation of cognitive functions (n = 13), and a study without a full-text (n = 1), a final selection of n = 27 studies was established (Fig. 1). During the search, several original articles or reviews involving non-psychiatric diagnoses with cognitive impairment were encountered. These include Parkinson’s disease [17–19] and fibromyalgia [20]. It was eventually decided not to include these studies into the review and focus solely on the psychiatric diagnoses. In the selected studies, the following parameters were assessed: study design, number of participants, participants’ diagnosis, mean age of participants, proportion of female participants, tACS parameters (device used, electrode placement, frequency and duration, stimulation intensity, and number of sessions), adverse effects of tACS, tested cognitive domains, scales used for cognitive testing, outcome of the studies, and presence of follow-up. Subsequently, the studies were divided based on the patients’ diagnoses (Table 1).

**Table 1:** Studies evaluating cognition changes after tACS in psychiatric disorders

Authors	Study	Functions assessed	Diagnosis (participant type)	Study design	Participant No.	Mean age	% female	Adverse effects	Electrode montage	Device	No. of sessions	Frequency	Intensity (peak-to-peak)	Duration	Scales	Cognitive outcomes	Other	Imaging methods or other biomarkers	Follow-up
Haller et al. [25]	Gamma transcranial alternating current stimulation in patients with negative symptoms in schizophrenia: a case series	Visual attention; word fluency; processing speed; WM	SCH	Case series	3	30.3 ± 6.3	100	Mild site discomfort; phosphenes	F3; F4	NeuroConn eldith^®^ DC-stimulator (Neuroconn, Ilmenau, Germany)	5 days/week for 2 weeks	40 Hz	2 mA	10 min, twice per day	RWT; TMT-A/B	Improvement in RWT, TMT-A/B	–	–	–
Haller et al. [24]	Gamma transcranial alternating current stimulation for treatment of negative symptoms in schizophrenia: report of two cases	Visual attention; word fluency; processing speed; WM	SCH	Case series	2	27	50	Phosphenes	F3; F4	NeuroConn eldith^®^ DC-stimulator (Neuroconn, Ilmenau, Germany)	5 days/week for 2 weeks	40 Hz	2 mA	20 min	RWT; TMT-A/B	Improvement in RWT and TMT-A/B	–	–	–
Sreeraj et al. [27]	Online theta frequency transcranial alternating current stimulation for cognitive remediation in schizophrenia: a case report and review of literature	WM; attention; processing speed	SCH	Case report	1	34	0	Mild itching	F3; P3	NeuroConn DC-stimulator Plus (Neuroconn, Ilmenau, Germany)	5 days	6 Hz	2 mA	20 min	N-back (0-, 1-, and 2-back) test; DSST	Improvement in N-back test and DSST	Improvement persisted during follow-up at 50 days	–	50 days
Sreeraj et al. [26]	Feasibility of online neuromodulation using transcranial alternating current stimulation in schizophrenia	WM	SCH	Case report	1	35	0	Mild pricking	F3; P3	NeuroConn DC-stimulator Plus (Neuroconn, Ilmenau, Germany)	Single session (2 sessions in total)	6 Hz; 40 Hz	2 mA; 1 mA respectively	20 min	N-back (0-, 1-, and 2-back) test; dual 2-back test	Improvement in N-back test and dual 2-back test in theta-tACS; no improvement in gamma-tACS	–	–	–
Hoy et al. [23]	Preliminary investigation of the effects of γ-tACS on working memory in schizophrenia	WM	SCH; SCHD	Single-blind, randomized, repeated-measures study	11	43.27 ± 10.02	55	Mild site discomfort	F3; supraorbital region	Elidth stimulator plus	Single session	40 Hz	2 mA	20 min	N-back task (2-)	No change in performance following gamma-tACS	–	–	–
Chang et al. [21]	Online left-hemispheric in-phase frontoparietal theta tACS for the treatment of negative symptoms of schizophrenia	WM; executive function; sustained attention; response inhibition; psychomotor speed; attention shift; selective attention	SCH; SCHD	Double-blind, randomized, sham-controlled trial	36	41.78 ± 8.84 in active tACS group	50	None	Two stimulators: 1st stimulator F1, F5, AF3, FC3 and CPz; 2nd stimulator P1, P5, CP3, PO3 and FCz	NeuroConn Eldith DC-stimulator plus (Neuroconn, Ilmenau, Germany)	5 days	6 Hz	2 mA	20 min, twice per day	Dual 2-back task; WCST; CPT-II; DS; FTT; TOL; CTT; stroop interference test	Significant improvement in dual 2-back task	Further predictors of tACS efficacy tested (HRV)	–	1 week, 1 month
Mellin et al. [22]	Randomized trial of transcranial alternating current stimulation for treatment of auditory hallucinations in schizophrenia	Verbal memory; WM; executive functions; attention	SCH; SCHD	Double blind, randomized, sham controlled trial	22	47 ± 9.72 in tACS group	32	Tingling, itching, burning; phosphenes	F3/Fp1 and T3/P3; Cz (return)	NeuroConn DC-stimulator Plus (Neuroconn, Ilmenau, Germany)	5 days	10 Hz	2 mA	20 min twice daily	BACS	Small effect size of tACS on BACS, no significant improvement	–	–	1 month
Benussi et al. [34]	Exposure to gamma tACS in Alzheimer’s disease: a randomized, double-blind, sham-controlled, crossover, pilot study	Episodic memory; associative memory	AD	Randomized, double-blind, sham-controlled, crossover, pilot study	20	71.9 ± 7.0	50	None	Pz (over medial parietal cortex and precuneus)	Battery-driven current stimulator (Brainstim, EMS, Italy)	Single session	40 Hz	3 mA	60 min	RAVL, FNAT	Significant improvement in RAVLT, FNAT	SAI increased, pilot study for Benussi et al. 2022	TMS for SAI	–
Benussi et al. [29]	Increasing brain gamma activity improves episodic memory and restores cholinergic dysfunction in Alzheimer’s disease	Episodic memory; associative memory	AD	Randomized, double-blind, sham controlled, crossover study	60	72.3 ± 7.0	51.7	None	Pz; deltoid muscle	BrainStim stimulator (E.M.S., Bologna, Italy)	Single session	40 Hz	3 mA	60 min	RAVL, FNAT	Significant effect of treatment on RAVL, FNAT	EEG recorded (significant association between gamma power over P3/P4 increase and RAVLT improvement; Further predictors of γ-tACS efficacy tested (significant association with ApoE genotype, baseline MMSE), SAI increased	EEG, TMS for SAI	–
		Executive functions; verbal fluency; visuo-spatial abilities	AD		12	NA	NA		Pz; deltoid muscle						DS backward; phonemic and semantic fluencies; TMT/A-B; clock drawing test	No significant effect			
		Episodic memory; associative memory	AD		12	NA	NA		F4; deltoid muscle						RAVL, FNAT	No significant effect			
Bréchet et al. [38]	Patient-tailored, home-based non-invasive brain stimulation for memory deficits in dementia due to Alzheimer’s disease	Delayed recall; memory; executive function; attention; visuospatial attention	AD	Case reports	2	79	0	Mild site tingling, burning	Left angular gyrus	BrainStim stimulator (E.M.S., Bologna, Italy)	5 days/week for 14 weeks	40 Hz	max. 2 mA on each electrode; 4 mA across all electrodes	20 min	MIS; MoCA	Improvement in MIS; MoCA	Improvement also in 3 month follow-up; tACS application at home	EEG only at the beginning	3 months
Liu et al. [39]	Transcranial alternating current stimulation combined with sound stimulation improves the cognitive function of patients with Alzheimer’s disease: a case report and literature review	Memory; visuo-spatial ability; executive function; attention; orientation; verbal recall	AD	Case report	1	73	100	None	DLPFC; contralateral supraorbital area	Transcranial electrical current stimulator (XPNS208-B, Suzhou Hypnos MD Co. Ltd., China)	5 days/week for 3 weeks	40 Hz	1.5 mA	20 min	CDR; ADAS-Cog; MoCA; MMSE; AVLT	Improvement in all used cognitive tests at the end of tACS sessions and also at 4 month follow-up	tACS combined with sound stimulation. Patient tested also at 4 month follow-up	–	4 months
Sprugnoli et al. [37]	Impact of multisession 40 Hz tACS on hippocampal perfusion in patients with Alzheimer’s disease	Episodic memory; verbal fluency	AD	Open-label study	10	72	40	Mild tingling, scalp irritation, visual changes, headache	Group 1: right temporo-frontal lobes, individually determined, close to T8; Group 2: bilaterally over teploral lobes	Battery-driven current stimulator (Starstim SS32, neuroelectrics, Barcelona, Cambridge)	5 days/week for 2 or 4 weeks	40 Hz	max. 2 mA on each electrode; 4 mA across all electrodes	60 min	Craft story 21 recall immediate and delayed; category fluency task	No significant improvement in memory and language tests	Primarily tested hippocampal perfusion and CBF. Significant CBF increase. CBF changes positively correlate with changes at craft story recall–delayed electrode position determined individually	EEG, CBF through MRI, PET (only for modelling)	–
					5				Group 3: bilaterally over temporal lobes		5 days/week for 4 weeks					No significant improvement in memory and language tests			
Zhou et al. [28]	Effects of 40 Hz transcranial alternating current stimulation (tACS) on cognitive functions of patients with Alzheimer’s disease: a randomised, double-blind, sham-controlled clinical trial	Memory; visuo-spatial ability	AD	Randomised, double-blind, sham-controlled trial	50	NA	NA	NA	Bilaterally over temporal lobes	Transcranial, London, UK	5 days/week for 6 weeks	40 Hz	2 mA	20 min	ADAS-Cog	Significant improvement in ADAS-Cog, MMSE	MMSE significantly higher at the end od tACS and 12-week follow-up	–	12 weeks
Moussavi et al. [36]	A novel program to improve cognitive function in individuals with dementia using transcranial alternating current stimulation (tACS) and tutored cognitive exercises	Auditory and visual memory; visual WM; immediate and delayed memory; recognition memory	Dementia	Open-label study	28, tACS in 19 participants	73.1 ± 1.8 in tACS group	32 in tACS group	Application site discomfort, pain	L-DLPFC; contralateral supraorbital area	Soterix medication tACS stimulator (model: 2001)	2 sessions/day, 5 days/week for 4 weeks	40 Hz	1.5 mA	30 min	WMS-IV	Significant improvement in WMS-IV in tACS + CT group	Simultaneous cognitive training, both groups improved post-intervention, tACS group improved also at 1 m FU	–	1 month
Kim et al. [40]	tACS as a promising therapeutic option for improving cognitive function in mild cognitive impairment: a direct comparison between tACS and tDCS	Inhibitory control; visual attention; processing speed; WM; attentional shift	MCI	Sham-controlled, double-blinded, repeated-measures, counterbalanced study	20	76.8 ± 3.2	65	None	F3; F4	MIND-D (YBRAIN, Republic of Korea)	Single session	40 Hz	2 mA	30 min	Stroop test; TMT-A/B	Significant improvement in Stroop and TMT-B	Compared also tDCS; increase in beta activity in the right frontal and parietal regions	EEG	–
Naro et al. [35]	Promising role of neuromodulation in predicting the progression of mild cognitive impairment to dementia	Global cognitive status; frontal functions; verbal and non-verbal memory; language; attention; executive functions	MCI; AD	Randomised, double-blind, sham-controlled crossover trial	35 AD; 25 MCI	72 ± 9 in AD; 73 ± 10 in MCI	55 in AD; 54 in MCI	NA	C3 or AF3-AF7 or AF3-F1 or FC3 or FCz; right mastoid	BrainStim stimulator (E.M.S., Bologna, Italy)	Single session	40 Hz to 120 Hz randomly	1 mA	10 min	DS; clock drawing test; RML; AM; CVF; LVF	Significant improvement in RML, DS, CVF, LVF, and AM in MCI patients in DMPFC-tACS	Tested tACS efficacy/responsiveness as predictor of MCI progression into AD	EEG	2 years
Alexander et al. [42]	Double-blind, randomized pilot clinical trial targeting alpha oscillations with transcranial alternating current stimulation (tACS) for the treatment of major depressive disorder (MDD)	Memory; executive function; attention; visuospatial attention; orientation	MDD	Double-blind, randomized, sham-controlled clinical trial	32	36.69 ± 13.08	84	Tingling; burning; sleepiness; headache; phosphenes	F3; F4; Cz (return)	Two devices: NeuroConn DC-stimulator plus (Neuroconn, Ilmenau, Germany)	5 days	10 Hz; 40 Hz	2 mA between F3/F4; 4 mA to Cz	40 min	MoCA	No significant improvement in MoCA	–	EEG	4 weeks
Haller et al. [43]	Gamma transcranial alternating current stimulation improves mood and cognition in patients with major depression	WM; verbal working memory; executive function; visual attention; processing speed	MDD	Randomized case series	6	35.7 ± 16.5	17	Phosphenes	F3; F4	NeuroConn eldith^®^ DC-stimulator (Neuroconn, Ilmenau, Germany)	5 days/week for 2 weeks	40 Hz	2 mA	Group 1: 10 min twice-daily, group 2: 20 min	RWT; TMT-A/B; N-back test	Improvement in verbal N-back test and TMT-A/B	Unable to prove significance levels due to small number of cases	–	–
Palm et al. [41]	Single session gamma transcranial alternating stimulation does not modulate working memory in depressed patients and healthy controls	WM	MDD	Double-blind, randomized, sham-controlled, crossover study	43 (22 MDD + 21 HA)	40.8 ± 13.71 in MDD group	68	No major adverse events	F3; F4	NeuroConn eldith® DC-stimulator (Neuroconn, Ilmenau, Germany)	Single session	40 Hz	2 mA	20 min	N-back task (1-, 2-, 3-)	No improvement in cognitive performance	Absence of effect could be caused by WM task training before tACS or little room for improvement in N-back task	–	–
Wilkening et al. [44]	Transcranial alternating current stimulation for the treatment of major depression during pregnancy	Visual attention; processing speed; WM	MDD in pregnancy	Case report	1	38	100	Phosphenes	F3; F4	NeuroConn eldith^®^ DC-stimulator (Neuroconn, Ilmenau, Germany)	2 weeks: 5 sessions in 1st week; 4 sessions in 2nd week	40 Hz	2 mA	20 min	TMT-A/B	Improvement in TMT-A/B	Pregnancy; improvement at 2-week FU	–	2 weeks
Haller et al. [45]	Gamma transcranial alternating current stimulation (γtACS) in obsessive–compulsive disorder: a case report	Visual attention; word fluency; processing speed; WM	OCD	Case report	1	28	100	NA	NA	NeuroConn eldith^®^ DC-stimulator (Neuroconn, Ilmenau, Germany)	5 days/week for 2 weeks	40 Hz	2 mA	20 min	RTW; TMT-A/B; N-back task (3-)	Improvement in RTW; TMT-A/B; N-back task (3-)	–	–	–
Amouzadeh and Sheikh [48]	Impact of transcranial alternating current stimulation on working memory and selective attention in athletes with attention deficit hyperactivity disorder: randomized controlled trial	WM; Inhibitory control; selective attention	ADHD	Randomized, sham-controlled trial	45	11.62 ± 0.35 in tACS group	33	NA	F3; supraorbital region	Stimulator by medinateb	5 days/week for 2 weeks	10 Hz	1 mA	15 min	Stroop test; N-back task	Improvement of selective attention	–	–	2 weeks
Dallmer-Zerbe et al. [47]	Transcranial alternating current stimulation (tACS) as a tool to modulate P300 amplitude in attention deficit hyperactivity disorder (ADHD): preliminary findings	Response inhibition; reaction time	ADHD	Single-blind, randomized, sham-controlled study	18	31.3 ± 9.89	39	No significant discomfort	C3, C4, CP3, CP4, P3, and P4; T7, T8, TP7, TP8, P7, and P8	NeuroConn DC-stimulator Plus (Neuroconn, Ilmenau, Germany)	Single session	Individually determined	1 mA	20 min	Go/NoGo task	No improvement in RT. Significant improvement in omission type errors	P300 ERP also tested, significant amplitude increase; phase-locked stimulation	EEG	–
Kannen et al. [46]	P300 modulation via transcranial alternating current stimulation in adult attention-deficit/hyperactivity disorder: a crossover study	Attention	ADHD	Single-blind, randomized, sham-controlled crossover study	20	28.55 ± 8.77	10	NA	C1/C2; C5/C6	NeuroConn DC-stimulator plus (Neuroconn, Ilmenau, Germany)	2 sessions (active, sham)	1 to 8 Hz individual frequency	1 mA	20 min	D2 attention test; visual oddball task	No significant improvement	P300 ERP also tested, no significant change	EEG	–
Daughters et al. [49]	Alpha-tACS effect on inhibitory control and feasibility of administration in community outpatient substance use treatment	Inhibitory control	SUD	Double-blind, randomized, sham-controlled trial	30	43.2 ± 7.74	30	Mild tingling and trouble concentrating	F3; F4; Cz (return)	“Pulvinar neuro XCSITE 100 stimulator (Chapel Hill, NC)”	Single session	10 Hz; 40 Hz	2 mA between F3/F4; 4 mA to Cz	40 min	Go/NoGo task	Significant improvement in inhibitory control in alpha-tACS group	–	–	–
McKim et al. [50]	Addiction history moderates the effect of prefrontal 10-Hz transcranial alternating current stimulation on habitual action selection	Action selection; perseverative errors	SUD	Double-blind, randomized, within-subjects, crossover study	37 (17 SUD + 20 HA)	39 ± 9 in SUD group	41 in SUD group	Burning; fatigue; itch; pain; warmth	F3; F4; Cz	NeuroConn DC-stimulator plus (NeuroConn, Ilmenau, Germany)	2 sessions (active, sham)	10 Hz	2 mA	30 min	HABIT task	Improvement in perseverance errors in SUD group–not significant	tACS effect significantly higher with length of SUD	–	–
Sabel et al. [51]	Non-invasive brain microcurrent stimulation therapy of long-COVID-19 reduces vascular dysregulation and improves visual and cognitive impairment	Attention; executive function; WM; flexibility; memory	PASC	Two case studies (cognition tested in one)	2	40 and 72	100	None	Near the forehead	SASm-neuromodulation device (SAVIR GmbH, Berlin, Germany)	13 and 10 respectively	NA	< 2 mA	30–45 min	TAP; AVLT	Improvement in cognitive domains in TAP and AVLT	–	–	–

*SCH* Schizophrenia, *SCHD* schizoaffective disorder, *AD* Alzheimer’s dementia, *MCI* mild cognitive impairment, *MDD* major depressive disorder, *OCD* obsessive–compulsive disorder, *ADHD* attention deficit hyperactivity disorder, *SUD* substance use disorder, *PASC* post-acute sequelae of Covid-19, *NA* not available, *FU* follow-up, *EEG* electroencephalography, *WM* working memory, *RWT* regensburg word fluency test, *TMT-A/B* trail making test A/B, *DSST* digit symbol substitution test, *WCST* Wisconsin card sorting test, *CPT-II* Connors’ continuous performance test-2nd edition, *DS* digit span test, *FTT* finger Tapping test, *TOL* tower of London, *CTT* color trails test, *BACS* brief assessment of cognition in schizophrenia, *RAVLT* rey auditory verbal learning test, *FNAT* face-name associations task, *MIS* memory index score, *MoCA* montreal cognitive assessment, *MMSE* mini-mental state examination, *CDR* clinical dementia rating, *ADAS-Cog* Alzheimer’s disease assessment scale-cognitive scale, *AVLT* auditory verbal learning test, *WMS-IV* Wechsler memory scale-fourth edition, *RML* reversal motor learning, *AM* attentive matrices, *CVF* category verbal fluency, *LVF* letter verbal fluency, *TAP* test of attentional performance, *AVLT* auditory verbal learning test, *RT* reaction time, *ERP* event-related potential, *HRV* heart rate variability, *SAI* short-latency afferent inhibition, *TMS* transcranial magnetic stimulation, *MRI* magnetic resonance imaging, *PET* positron emission tomography, *DLPFC* dorsolateral prefrontal cortex, *CBF* cerebral blood flow, *HABIT* hidden association between images task

## Results

### Schizophrenia

Seven of the reviewed studies included patients with schizophrenia (SCH) or schizoaffective disorder. Three of them were randomized control trials, two with multiple-session tACS, and one with a single-session application. Four studies were case reports or case series, out of which three used multiple-session tACS, and one was a single-session.

#### Randomized control trials

One study [21] used 10 sessions of 6 Hz left-side fronto-parietal stimulation. The primary goal was to reduce negative symptoms, which was accomplished. The stimulation also improved working memory (WM) performance, which lasted at one-week and one-month follow-up. This study also aimed to find a biomarker of therapeutic response, with heart rate interval change during the WM task successfully predicting the patients’ tACS-responsiveness. Another study [22] used five sessions of 10 Hz frontal and left-side temporo-parietal stimulation in order to attenuate the severity of auditory hallucinations. The participants were also tested with the Brief Assessment of Cognition in Schizophrenia (BACS), without any significant improvement. The third randomized trial [23] used 40 Hz stimulation over F3 area in a single session, which aimed at improving WM, without any significant changes.

#### Case reports and case series

Two studies [24, 25] used 10 sessions of 40 Hz stimulation over F3/F4 in two and three patients respectively and found improvements in visual attention, word fluency, processing speed, and WM. One case report [26] also applied 40 Hz stimulation but over P3 area and in a single session, without any influence on cognition. However, a single session of 6 Hz stimulation over F3 resulted in WM improvement. Another case report by the same team [27] applied five sessions of 6 Hz stimulation over F3/P3, similar to [21], resulting in WM improvement observable also at the 50-day follow-up.

### Dementia

Eight studies included patients with dementia: seven out them were focused solely on AD, and one covered dementia in general. Four studies were randomized control trials, out of which one was a multi-session study and three were single-session. Out of the other studies, two were open-label (both multi-session), and two were case reports (also both multi-session).

#### Randomized control trials

A double-blind randomized control study [28] applied 30 stimulations of 40 Hz bitemporally with a significant improvement in memory and visuospatial abilities. Patients performed better in the Alzheimer’s Disease Assessment Scale–Cognitive subset (Adas-Cog) and Mini–mental state examination (MMSE), with improved MMSE performance observable in a 12-week follow-up. Another double-blind randomized study [29] used a single session of 40 Hz over Pz, which improved episodic and associative memory, measured by Rey Auditory Verbal Learning Test (RAVLT) immediate and delayed recall. This study also aimed to uncover potential predictors of post-tACS improvement. Significant association was found in ApoE ε4 non-carriers and baseline MMSE score (the higher the baseline MMSE, the greater the post-tACS response). Resting-state electroencephalography (EEG) analysis also found a significant association between the increase in gamma frequencies over P3 and P4 and a post-tACS delayed recall improvement. Short-latency afferent inhibition (SAI), which evaluates the cholinergic transmission, was also assessed using transcranial magnetic stimulation (TMS). TMS (either single-pulse TMS [30] or paired-pulse TMS [31]) is a non-invasive technique which measures the change in motor evoked potentials, assessing the activity of various neurotransmitter systems and overall cortical excitability [32]. SAI was decreased (i.e. improved closer to values found in healthy controls [33]) post-tACS, which also correlated with delayed recall in RAVLT improvement. Additionally, the influence of this protocol on different cognitive functions (executive functions, verbal fluency, and visuospatial abilities) and efficacy of the same frequency but different electrode placement (F4) on episodic and associative memory were evaluated. None of these assessments yielded any significant results, therefore confirming the specificity of electrode placement and the corresponding affected cognitive functions. This study was preceded by a pilot study by the same team [34], with identical tACS parameters and a smaller group of patients, which too found improvement of episodic and associative memory, and a significant decrease in SAI. One study [35] included patients with both AD and mild cognitive impairment (MCI) in a single session of various stimulation frequencies (40 Hz to 120 Hz randomly) with various electrode placement (C3, AF3-AF7, AF3-F1, FC3, or FCz). In a subset of AD patients, no significant behavioural or EEG changes were observed.

#### Open-label studies and case reports

One open-label study [36] applied 40 stimulations of 40 Hz over L-DLPFC and contralateral SOA in patients with dementia of unspecified origin. In this study, all patients underwent cognitive training (CT), with one group also receiving tACS as an enhancement of CT. This study did not find any significant memory improvement. An open-label study [37] applied 10 stimulations of 40 Hz fronto-temporally on the right side, or bitemporally, without significant improvement in cognitive tests. However, significant increase in cerebral blood flow (CBF), measured by arterial spin labelling through magnetic resonance imaging (MRI), was observed post-tACS and correlated with gamma band power increase under the T7/8-P7/8 electrodes in resting-state EEG. One case study [38] used home-based tACS application, with 70 stimulations of 40 Hz over left angular gyrus. This protocol led to improvement in memory, executive function, and attention, which persisted also in a 3-month follow-up. Furthermore, this exceptionally long protocol provided data on treatment tolerability and patient compliance, with 100% adherence (no missed stimulations) and no serious side effects or adverse events. Another case report [39] applied 15 stimulations of 40 Hz over DLPFC and contralateral supraorbital area (SOA), which resulted in improvement of memory, visuospatial abilities, executive function, attention, orientation and verbal recall. This improvement also persisted in a 4-month follow-up.

### Mild cognitive impairment

The effect of tACS in MCI patients was evaluated in two double-blind randomized control studies, both applying a single-session tACS.

#### Randomized control trials

One study [40] applied a single session of 40 Hz tACS over F3/F4, with an improvement in inhibitory control, visual attention, processing speed, and WM. In resting-state EEG analysis, beta activity in the right frontal and parietal regions was increased post-tACS. A previously mentioned study [35] with a single session of various frequencies and electrode montages (EMs) included both AD and MCI patients. In a subset of MCI patients, stimulation over AF3-AF7 and AF3-F1 resulted in improvement of frontal functions, memory, language, attention and executive functions (tACS-responders). Moreover, subsequent resting-state EEG measurement showed a gamma band power increase in healthy controls and tACS-responders with MCI over all electrodes groups after DLPFC stimulation, over frontal-central electrodes after DMPFC tACS, and over central electrodes after M1 tACS. The behavioural response and electrophysiological changes post-tACS were able to predict the risk of progress into dementia, where tACS non-responders converted into dementia in a 2-year follow-up, whereas tACS responders’ status remained stable.

### Major depressive disorder

Four studies evaluated the tACS-induced cognitive functions change in major depressive disorder (MDD). Two were randomized control trials, one multi-session and one single-session. The other studies include one case series (multi-session), and one was a case report (multi-session).

#### Randomized control trials

One of the double-blind randomized control trials (RCT) [41] used a single session of 40 Hz over F3/F4 and failed to find significant results. Another study [42] applied five sessions of 10 Hz or 40 Hz tACS over F3, F4, and Cz, with cognition tested through Montreal Cognitive Assessment (MoCA), and also did not find any significant results. Although no significant change was found in the MoCA test, significantly reduced resting-state alpha oscillations over the left frontal regions were found in EEG.

#### Case reports and case series

A randomized case series [43] used 10 sessions of 40 Hz tACS over F3/F4 and found improvement in WM, visual attention and processing speed, albeit the results did not reach significance. One case report [44] applied the same protocol in a pregnant woman, with an improvement in visual attention, processing speed and WM, present also at a 2-week follow-up.

### Obsessive–compulsive disorder

#### Case report

To date, only one case report exists that evaluated cognitive functions post-tACS in a patient with obsessive–compulsive disorder (OCD). In this case report [45], 10 stimulations of 40 Hz resulted in improvements of attention, word fluency, processing speed and WM.

### Attention deficit hyperactivity disorder

Three studies included patients with attention deficit hyperactivity disorder (ADHD). All of them were randomized control trials, two were single-session, and one used a multi-session application.

#### Randomized control trials

One study [46] applied a single session of 1 to 8 Hz over C1/C2 and C5/C6, without any cognitive improvement. This study also evaluated the P300 over centro-parietal regions, based on the hypothesis of reduced P300 generated by low frequency event-related oscillations (EROs), without any significant change. Similarly, another study [47] applied a single session of individually determined delta/theta frequency bilaterally temporo-parietally, with subsequent P300 evaluation. TACS in this study was phase-locked by presenting the P300-inducing stimulus at such latency that the response matched the tACS phase. This protocol led to a significant P300 amplitude increase and omission-type errors improvement. The third study [48] used 10 stimulations of 10 Hz in ADHD athlete children and found a significant improvement in WM.

### Substance use disorder

Two studies evaluated the effect of tACS in patients with SUD. Both were randomized control trials with a single-session tACS.

#### Randomized control trials

One study [49] focused on the inhibitory control changes. The authors found an improvement after a single session of 10 Hz tACS over F3, F4 and Cz, but not after 40 Hz tACS. Another study [50] investigated the effect of a single session of 10 Hz tACS over F3, F4 and Cz on habitual action selection and perseverance errors. Interestingly, although this single session increased the number of errors in healthy controls, SUD patients’ performance improved. They exhibited a decrease of perseverance errors, with the size of this decrease correlating with SUD duration – i.e. the longer the SUD, the greater the perseverance errors reduction.

### Post-acute sequelae of COVID-19

#### Case report

One case study [51] investigated the effects of tACS in relation to COVID-19. A patient with neuropsychiatric post-acute sequelae of COVID-19 (PASC) underwent 13 tACS sessions of undisclosed frequency over the forehead. This protocol resulted in improvements of attention, memory, executive functions and WM.

### Risk of bias

Risk of bias was evaluated in all listed studies. Twelve studies other than randomized control trials (i.e., case reports, case series and open-label studies) were labeled as “High risk” due to a lack of control group and blinding [24–27, 36–39, 43–45, 51]. Randomized control trials (RCT) were assessed according to the Cochrane database RoB 2 tool [52], with the assessed domains being randomization process, deviations from intended interventions, missing outcome data, measurement of the outcome, selection of the reported result and overall bias. Out of 15 RCT, three were evaluated as “Some concerns” due to single-blind design [23, 46, 47], two due to insufficiently described statistical analysis [21, 35], one due to unsuccessful blinding integrity [41], and one due to insufficiently described blinding procedure [48]. Eight studies were considered “Low risk” [22, 28, 29, 34, 40, 42, 49, 50].

## Discussion

We reviewed 27 studies that applied tACS in patients with psychiatric diagnoses and evaluated change in cognitive functions. The diagnoses in question include SCH and schizoaffective disorder, AD/MCI, MDD, OCD, ADHD, and PASC. If a particular study measured neurophysiological changes, besides the behavioural changes, these effects were also noted. Here, we list the range of CI and EOs pathologies and subsequently attempt to relate and integrate them with the reviewed studies to form a comprehensive progression from pathological processes to possibilities of their improvement. Both phenomena are most extensively described in SCH and AD/MCI, with corresponding highest numbers of relevant tACS studies.

None of the studies reported a serious adverse event or serious tACS side effect. The treatment was well-tolerated, with most frequently reported side effects being mild site discomfort, itching or tingling sensations, headache, phosphenes or fatigue.

### Schizophrenia

Cognitive deficit represents one of the major symptom groups in SCH patients, with 80% suffering from some kind of CI [53]. Described impaired domains include all types of memory (WM, short-term and long-term memory, episodic and semantic memory), executive functions, processing speed, verbal fluency and social cognition (for full review see [54]). There is little decline of CI over time and little to no difference of CI between medicated and unmedicated patients. Cognitive deficit appears before the first psychosis episode and remains stable [55].

Pathological EOs in SCH patients have been found during various cognitive tasks as well as at resting state (for full review see [56]). Among others, a gamma-band reduction has been observed in resting-state, induced and evoked oscillations [56–58]. Impairment of induced gamma and beta-band synchronization has been associated with functional disconnectivity [56]. Specifically, impairment of gamma-band oscillations over frontal regions is a result of reduced interneuron inhibition of pyramidal cells [56, 59]. Layer 3 pyramidal neurons (L3PN) of the DLPFC exhibit smaller volumes and lower dendritic spine density [60], resulting in hypoactivity of this layer [61]. The diminished excitatory activity of its neurons decreases through a feedback loop the activity of parvalbumin basket cells (PVBC), which function as inhibitory interneurons for L3PN. This might be interpreted as a compensatory effect to maintain excitatory/inhibitory balance [62]. However, a correct PVBC inhibition, mediated by GABA, results in a large-scale synchronous hyperpolarization and subsequent depolarization at gamma frequency, creating gamma-band oscillations [62]. These gamma-band oscillations over the prefrontal cortex are a correlate of performing a WM task, with a gamma power increase in higher load WM task [63–66]. WM-related gamma-band reduction (in amplitude and frequency) is associated with poor WM performance, and gamma-band amplitude positively correlates with GABA level in DLPFC [67]. Therefore, the microcircuit of L3PN and PVBC in DLPFC generates gamma-band oscillations and by extension forms the basis of WM [68]. Its disruption then propagates across various structures [69] partaking in WM functioning [68]. Imposing gamma frequency oscillations by tACS over DLPFC might therefore affect the disrupted process directly.

For SCH, tACS has a greater effect when administered in multiple sessions (five or more), while studies applying a single-session tACS [23, 26] failed to find a significant result. This might be due to inability of one stimulation to produce a substantial change in cognition, or due to interference with the underlying pathology [23]. The electrode montage and frequencies with most pronounced results are F3/P3 at theta and F3/F4 at gamma frequency. Therefore, it seems that tACS targeted at L-DLPFC (corresponding to F3 electrode) improves cognition in SCH. This is in line with studies that describe macroscopic and microscopic abnormalities in DLPFC in SCH [61].

Some studies suggest that not only DLPFC gamma-band oscillations but also fronto-medial theta-band oscillations are a part of the WM mechanism [70]. This might explain good results of theta frequency tACS over F3/P3. On the other hand, one multi-session study, that did not reach significant results [22], measured the change in cognition through BACS, which may not be sensitive enough to detect more subtle changes, in WM or other.

All these results are integrated in a theory describing WM as an interplay and coupling between different frequencies and structures [71, 72]. So-called cross-frequency coupling (CFC), i.e., a relation between two distinct frequencies in phase or amplitude, has been described in various cognitive processes [73]. Theta-gamma phase-amplitude coupling, a CFC subtype, is specifically related to WM performance [1, 74]. One theory states that the number of gamma oscillations nested in the peak of a theta wave is responsible for WM item organisation [75]. Such WM-related EOs entrainment in healthy adults results in improved WM performance [76]. Moreover, this theta-gamma coupling has been shown to be impaired and decreased in SCH patients during WM task, whereas in healthy controls it corresponds with increasing WM load [77]. On the other hand, resting state theta-gamma coupling is increased in first-episode psychosis patients, which correlates with better cognitive performance, suggesting a compensatory hyperactivation, before eventual theta-gamma coupling decrease [78]. Therefore, theta-gamma tACS and CFC-oriented stimulation might be a promising direction for future tACS application.

### Dementia

According to the diagnostic and statistical manual of mental disorders, 5th edition (DSM-V) [79], dementia, or major neurocognitive disorder, represents a group of disorders involving a cognitive decline that interferes with basic daily functioning. In its most common pathological unit, AD, disruptions of memory (episodic, associative and WM), planning abilities and executive functions are present [80, 81].

Multiple changes have been found in EOs in patients with AD. A general slowing (a reduction of fast alpha, beta and gamma-band oscillations and increase of slower delta and theta-band oscillations) in resting-state EEG has been well-documented [82]. Further pathologies include a delayed gamma event-related latency in parietal regions in the visual oddball paradigm (VOP) [83] but also increased gamma connectivity in VOP [84] and reduced theta-gamma coupling during a WM task [85] (for full review see [86]). Conflicting results in resting-state EEG [82] may be overcome in combined TMS-EEG studies by evaluating TMS-induced perturbations. Increase in gamma-band power as a result of TMS and lack of this increase over left DLPFC in AD patients allowed them to be distinguished from healthy age-matched controls, also predicting a cognitive decline in a 24-week follow-up [87].

Nearly all studies with AD patients used 40 Hz frequency tACS, which is in line with previous research achieving gamma entrainment through sensory stimuli (visual or auditory). Both stimulation types resulted in improvement of spatial and recognition memory [88] and also amyloid and tau-protein reduction and changes in microglial response in a mouse model [88, 89]. In the first similar human case series, gamma-tACS resulted in a significant decrease of tau-protein in the temporal lobe [90]. Similar to SCH, gamma oscillations are generated by GABA-mediated inhibitory interneuron spiking [91]. During the AD progression, resting-state gamma oscillation power increases at first as a result of compensatory mechanisms, subsequently decreasing, when these mechanisms are insufficient to overcome the amyloid burden and neurodegeneration level [92]. Gamma-tACS might therefore serve both as an enhancement of these compensatory mechanisms and as a method to induce protein clearance [93]. As for the electrode montage, DLPFC tACS and parietal, and left angular gyrus tACS yielded a cognition improvement. Bitemporal tACS improved cognition after 30 sessions [28], but not after 20 sessions [37], further advocating for protocols with more sessions, however, differences in cognitive tests may also play a role. Positive results with largely different electrode montage might be explained by a variety of found behavioural, structural and functional pathologies. However, specific electrode montages result into changes in specific cognitive domains, e.g. tACS over Pz affects episodic and associative memory, but not executive functions of visuo-spatial abilities [29]. Tau-protein and amyloid build-up burden can be found mostly in temporal, frontal and parietal lobes, respectively [94]. DLPFC, on the other hand, exhibits plasticity impairments [95]. Therefore, multiple electrode montages are suitable candidates for tACS, each yielding different behavioural results [29].

### Mild cognitive impairment

MCI is a stage of cognitive decline where patients perform worse than healthy adults in neuropsychological tests but are able to perform basic daily activities [79]. The largest group of MCI patients (about 50%) have an underlying AD pathology, with eventual progression into AD at a conversion rate of 5–17% per year [96]. However, MCI may also remain stable or progress into different dementia types, such as fronto-temporal dementia or Lewy body dementia [97]. This heterogeneity in underlying pathological processes may cause discrepancies in studies evaluating EOs or tACS treatment.

EEG studies describe differences in event-related synchronization and desynchronization (ERS/ERD) in MCI patients compared to healthy older adults in multiple frequency bands and localizations [98], with theta-band ERD discriminating between stable and progressive MCI [99]. Event-related oscillations in delta (fronto-centrally) and theta-band (fronto-parietally on the right side) also decline in MCI patients, with spatial spreading of this decline during progression into AD [100].

Both studies with MCI patients used gamma-tACS, one study at 40 Hz (applied at F3/F4) and the other at random frequency in range 40–120 Hz (applied at various electrodes). Although both studies reached significant improvement in multiple cognitive domains, they only used a single session protocol and therefore cannot be considered as long-term treatment protocols. However, as MCI is a transitional state in the healthy aging-dementia continuum, it is important to determine diagnostic markers able to stratify stable patients and patients with risk of progression into dementia. This was successfully attempted, with gamma-tACS responders being less likely to convert into dementia, which may be explained by a remaining cognitive reserve able to produce a tACS response [35]. EEG studies evaluating other frequency bands also found alpha ERS/ERD differences between healthy adults and MCI patients, which changed with task difficulty [98]. Attempts at affecting or entraining these frequency bands might be interesting for future studies.

### Major depressive disorder

Although it is not the main symptom group nor part of the diagnostic criteria, CI has been described in MDD [101]. Certain deficits have been documented in executive function, set shifting, inhibition, WM, verbal processing, attention, learning and memory [102]. These deficits (mainly in executive function) have been associated with impaired social and occupational functioning [102, 103].

As for the pathological oscillations, gamma-band power is increased frontally and temporally both at rest and during mental arithmetic counting test and spatial imagination task; however, it also shows decreased coherence and a reduced number of interactions [104]. In other neuroimaging studies, hypoconnectivity in DLPFC as a part of the frontoparietal cognitive control network is described [101]. A gamma-tACS protocol combined with intermittent theta-burst TMS enhanced TMS-induced gamma band oscillations in DLPFC in healthy adults [105], creating a possible targeted intervention for MDD patients. Alpha power asymmetry in frontal regions distinguishes between MDD patients and healthy adults, although it is not directly connected with CI [106].

In MDD patients, most studies used 40 Hz tACS. Overall, the number of studies and participants is too small to draw reliable conclusions. One protocol yielding positive results was gamma-tACS over F3/F4, which might be explained as targeting the hypoconnectivity in DLPFC and by extension the frontoparietal network [107]. Interestingly, the same frequency and electrode montage did not have any effect in a single session, which further encourages the use of multiple-sessions protocols. Appropriate cognitive tests also have to be used, as seen in [42], where the MoCA screening test was used. Patients reached the normal range of tests results at baseline, leaving little to no room for potential improvement.

### Obsessive–compulsive disorder

OCD patients exhibit a great level of phenotype heterogeneity, with various deficits across the cognitive domains [108]. Most notable impairments are in response inhibition/inhibitory control and planning [109, 110]. Studies offer mixed results in set shifting, verbal fluency, attention, non-verbal memory and visuospatial abilities. WM is comparable to healthy adults during low cognitive load tasks but worsens with higher loads [108].

Pathological EOs have been documented in frontal and occipital areas during WM tasks in OCD patients. Alpha ERD reduction have been described during encoding phase of WM task [111], which can be explained by an alpha power increase connected to excessive effort to inhibit intrusive thoughts and stronger alpha phase locking in OCD patients, which may be linked to excessive attentional processing. Alpha ERD reduction has also been described in the retrieval phase of memory task in a magnetoencephalography study [112]. Furthermore, aberrant beta-gamma phase amplitude coupling in fronto-central regions has been described [113].

One case report involving an OCD patient, which found an improvement with 40 Hz tACS over F3/F4, cannot be reliably explained by a particular underlying pathology. The gamma frequency protocol is used in most studies listed in this review and is in line with other diagnoses. Therefore, future research might elicit the exact mechanism of tACS effect on OCD pathology.

### Attention deficit hyperactivity disorder

ADHD patients suffer from an impairment of attention. However, deficits in other cognitive domains are also documented, namely in memory and executive function [114].

The elevated theta/beta ratio (TBR), although initially seen as a promising ADHD biomarker [115], was later not confirmed as specific [116, 117]. Recent studies offer mixed results on its possible origin and use [118, 119]. Resting state alpha power changes over frontal regions have also been documented in ADHD patients, with its normalization through neurofeedback correlating with improvement in inhibitory control task [120]. Although certain frequency powers may predict the treatment response [121], no definite connection can be made between particular power bands and impaired cognitive functions. P300, an attention-related event-related potential (ERP), shows a decrease in amplitude and a longer latency [122, 123]. Some studies suggest that the P300 ERP may be generated by delta and theta event-related oscillations (EROs) [124, 125].

In ADHD patients, although the listed studies may be related to certain documented pathological findings, the number of studies is too small, and the protocols with positive results may serve only as a guide for further experiments. It seems that individual phase-locking of tACS results in cognitive improvement [47], unlike a non-individualized protocol [46]. This phase-locking is feasible with a visual stimulus without the need for a closed-loop system. However, multiple-sessions protocols have to be applied to evaluate a potential long-term effect. A 10-session 10 Hz protocol resulting in a cognitive improvement is backed with some specific pathological basis [120] and therefore represents a viable direction of future research. Some parallels can be found in a study with healthy participants, in which an increase in alpha power was found over frontal eye field while performing a visual inhibitory control task [126], i.e., an alpha-power increase in the inhibited area. In ADHD patients, DLPFC seems to be hypoactive with a compensatory increased activation of deeper striatal regions [127].

### Substance use disorder

Impairment of cognitive control has been described as a risk factor in patients with substance use disorder [128, 129]. Adolescents with low cognitive control exhibit a more pronounced insula activation, connected with anticipation of reward, after substance use [130]. Inhibitory control, which is a subunit of cognitive control, is also impaired in SUD patients [131, 132]. Poor performance in inhibitory control tasks is connected with lower N2 amplitude [133]. On the other hand, higher N2 and lower resting state beta power are described as biomarkers of abstinence and good treatment outcomes [134]. Further pathologies have been described mainly in the ERP group (for comprehensive reviews, see [135, 136]).

In SUD patients, studies report findings of pathological ERPs, with a very limited amount of information about underlying EOs (both resting-state and event-related). The available studies found an improvement in inhibitory control and a decrease in perseverance errors in a single session of 10 Hz alpha tACS. However, for potential clinical use, multiple-session protocols are needed. Considering that inhibitory control is impaired similarly as in ADHD patients and has also been positively modified with 10 Hz tACS, a similar pathophysiological process and mechanism of action might be employed.

### Post-acute sequelae of COVID-19

An increasing phenomenon is CI following Covid-19 infection [137]. Reportedly, approximately 22% of patients suffer from a CI 12 weeks after Covid-19 diagnosis [138]. The impaired cognitive domains are attention, executive function, memory, verbal fluency and processing speed [139].

Despite multiple studies evaluating pathological EEG in Covid-19 patients [140, 141], to the authors’ knowledge there are not yet any studies concerning pathological EOs in relation to CI following Covid-19 infection.

TACS, among other NIBS methods [142, 143], was used experimentally in amelioration of cognitive symptoms of PASC due to a rising number of these cases. Its potential mechanism of action might be working against neuroinflammation and promoting protein clearance, seen with gamma-tACS in AD [91, 93]. Currently, the use of tACS in PACS remains purely empirical.

### Limitations

A limitation preventing the introduction of tACS into clinical practise is the knowledge gap concerning its long-term effect. In the studies listed in this review, the presence of a follow-up measurement is irregular (in 11 out of 27 studies). From case reports and randomised control trials applying a larger number of tACS sessions and a follow-up [27, 28, 38, 39], it seems possible to achieve long-term effect in various diagnoses. However, due to a limited overall number of studies, it is currently not possible to draw definite conclusions or determine closer details, e.g., how many sessions are needed for a sustained long-term effect to occur, how long will this effect last or if it is possible to maintain this effect by an occasional tACS application. Furthermore, the use of imaging methods in follow-up is needed to determine the exact mechanisms of long-term change occurrence.

Another limitation is an unclear connection between described pathological EOs and used tACS protocols. Most commonly, 13 of the 27 reviewed studies used electrode montages F3/F4, F3/F4/Cz, F3/Supraorbital region or DLPFC/Supraorbital region. Other montages included frontoparietal, parietal, frontal, bitemporal, over left angular gyrus, central, interchangeable, or individually determined. However, only a part of the listed studies can be directly connected with a particular pathology, mainly in SCH and AD/MCI. In other diagnoses, the number of studies investigating EOs (whether they are resting-state or event-related) in relation to CI is small. Therefore, the used tACS protocols are chosen based on previous results in other diagnoses or studies with healthy participants and not directly in relation to the underlying pathology. Subsequently, if a tACS protocol improves the CI, a possible pathophysiological connection has to be established post-hoc.

### Future directions

Potential future directions in clinical and pathophysiological domains may be derived from specific methods used in case reports and promising results in studies with healthy participants. In the clinical part, future research should address home-based tACS administration, individually adjusted tACS, tACS combined with other modalities, selecting tACS-responsive population, and tACS response as a predictive marker. For the pathophysiological part, tACS combined with imaging methods and tACS with complex waves are of great interest.

Home-based tACS offers a possibility of easy application in a comfortable setting. Devices with impedance control and inter-stimulation interval control allow for application by non-professional personnel after training. This enables distribution to a broad spectrum of patients, including those who are immobile, marginalized, or unable to travel frequently to a health facility.

Individually adjusted tACS involves individually determined electrode montage [37], frequency [46, 47], and phase-locking through closed-loop [144] or stimulus-locked tACS [47]. During individually determined electrode montage, the stimulation electrodes are chosen according to the precise location of a given EEG parameter (e.g., highest frequency amplitude) or according to the model of electrical field distribution [145], which compensates for the inter-individual anatomical differences. Similarly, tACS frequency can be adjusted according to the most prevalent frequency in the desired location. Phase-locked tACS enables the synchronization of tACS with EOs, providing their enhancement [144]; however, the effectivity varies across studies [146, 147]. These methods shift tACS protocols from predetermined to patient-tailored.

Combination with other modalities, such as with cognitive training, rTMS or sound stimulation, was already employed in some studies listed in this review [36, 39]. Cognitive training alone is already used in patients with SCH or AD [148–150]. Multimodal stimulation by combination with other stimulation types allows for more targeted and pronounced effect [151]. Sound stimulation can periodically induce ERPs/EROs in neuronal populations processing the sensory input, which are then enhanced by tACS [152]; however, a clinical use of this method requires further investigation.

For a successful introduction and use of tACS in psychiatric clinical practice, a selection and description of the tACS-responsive population, i.e., patients, who would benefit from tACS treatment, is needed. The factors that can affect the individual responsiveness to tACS might include anatomical or functional differences measured in MRI or EEG, level of cortical excitability, or cognitive reserve [93, 153]. These potentially predictive biomarkers for treatment response should be examined in interventional studies, with detailed examinations and stratification of responders from non-responders, first in post-hoc analyses, with subsequent confirmation in targeted studies.

Use of tACS response as a predictive marker has been described in this review [35]. Incorporation into the diagnostic protocol may help stratify patients according to their prognosis and initiate a swift intervention.

With concurrent use of imaging methods, particularly MRI, during tACS, we are able to directly observe the induced perturbations and effect on brain networks [154]. MRI-compatible tACS machines may be used in a resting state but also during task performance, depicting in real-time the tACS-induced effect [155].

Recently, tACS with complex superimposed waves has been used in studies with healthy participants. In this stimulation type, faster waves are nested in slower waves, resembling the endogenous cross-frequency coupling. In healthy participants, superimposed-wave tACS results in a WM improvement [76], or cognitive control decrease [156]. Cross-frequency coupling seems to be impaired in various psychiatric disorders [157]; therefore, this type of tACS might constitute a promising intervention.

## Conclusions

We described the current state of knowledge of tACS use in cognitive function improvement in various psychiatric diagnoses, connected it with described pathophysiological processes and outlined possible future directions of tACS use. Besides randomized control trials, we also included case reports/case series and used their results (in relation to the known pathological processes) as a hypothesis-generating point for further research.

TACS is a safe and well-tolerated intervention method, potentially capable of yielding a lasting cognitive improvement in patients across various psychiatric disorders. It is also feasible for home-based application. Stimulation parameters may be variously individualized to accommodate for patients’ anatomical and functional differences. Therefore, the ease of administration, safety, and possibility of individualized treatment are the greatest strengths of this method and its possible future introduction into clinical practice. Still, its limitations lie in significant knowledge gaps regarding its effectiveness, and further research is needed to confirm the specific effects of particular protocols in each disorder.

## References

[1] Wischnewski M, Alekseichuk I, Opitz A (2023). Neurocognitive, physiological, and biophysical effects of transcranial alternating current stimulation. Trends Cogn Sci.

[2] Liu A, Vöröslakos M, Kronberg G (2018). Immediate neurophysiological effects of transcranial electrical stimulation. Nat Commun.

[3] Alekseichuk I, Falchier AY, Linn G (2019). Electric field dynamics in the brain during multi-electrode transcranial electric stimulation. Nat Commun.

[4] Elyamany O, Leicht G, Herrmann CS, Mulert C (2021). Transcranial alternating current stimulation (tACS): from basic mechanisms towards first applications in psychiatry. Eur Arch Psychiatry Clin Neurosci.

[5] Buzzell GA, Barker TV, Troller-Renfree SV (2019). Adolescent cognitive control, theta oscillations, and social observation. Neuroimage.

[6] Karakaş S (2020). A review of theta oscillation and its functional correlates. Int J Psychophysiol Off J Int Organ Psychophysiol.

[7] Klimesch W (1999). EEG alpha and theta oscillations reflect cognitive and memory performance: a review and analysis. Brain Res Brain Res Rev.

[8] Mably AJ, Colgin LL (2018). Gamma oscillations in cognitive disorders. Curr Opin Neurobiol.

[9] Sadaghiani S, Kleinschmidt A (2016). Brain networks and α-oscillations: structural and functional foundations of cognitive control. Trends Cogn Sci.

[10] Fernandez-Ruiz A, Sirota A, Lopes-Dos-Santos V, Dupret D (2023). Over and above frequency: gamma oscillations as units of neural circuit operations. Neuron.

[11] Klink K, Paßmann S, Kasten FH, Peter J (2020). The modulation of cognitive performance with transcranial alternating current stimulation: a systematic review of frequency-specific effects. Brain Sci.

[12] Lee TL, Lee H, Kang N (2023). A meta-analysis showing improved cognitive performance in healthy young adults with transcranial alternating current stimulation. NPJ Sci Learn.

[13] Fusco G, Cristiano A, Perazzini A, Aglioti SM (2022). Neuromodulating the performance monitoring network during conflict and error processing in healthy populations: Insights from transcranial electric stimulation studies. Front Integr Neurosci.

[14] Nissim NR, McAfee DC, Edwards S (2023). Efficacy of transcranial alternating current stimulation in the enhancement of working memory performance in healthy adults: a systematic meta-analysis. Neuromodulation J Int Neuromodulation Soc.

[15] Booth SJ, Taylor JR, Brown LJE, Pobric G (2022). The effects of transcranial alternating current stimulation on memory performance in healthy adults: a systematic review. Cortex J Devoted Study Nerv Syst Behav.

[16] Page MJ, McKenzie JE, Bossuyt PM (2021). The PRISMA 2020 statement: an updated guideline for reporting systematic reviews. BMJ.

[17] Del Felice A, Castiglia L, Formaggio E (2019). Personalized transcranial alternating current stimulation (tACS) and physical therapy to treat motor and cognitive symptoms in Parkinson’s disease: a randomized cross-over trial. NeuroImage Clin.

[18] Cole RC, Okine DN, Yeager BE, Narayanan NS (2022). Neuromodulation of cognition in Parkinson’s disease. Prog Brain Res.

[19] Goodwill AM, Lum JAG, Hendy AM (2017). Using non-invasive transcranial stimulation to improve motor and cognitive function in Parkinson’s disease: a systematic review and meta-analysis. Sci Rep.

[20] Bernardi L, Bertuccelli M, Formaggio E (2021). Beyond physiotherapy and pharmacological treatment for fibromyalgia syndrome: tailored tACS as a new therapeutic tool. Eur Arch Psychiatry Clin Neurosci.

[21] Chang C-C, Huang CC-Y, Chung Y-A (2021). Online left-hemispheric in-phase frontoparietal theta tACS for the treatment of negative symptoms of schizophrenia. J Pers Med.

[22] Mellin JM, Alagapan S, Lustenberger C (2018). Randomized trial of transcranial alternating current stimulation for treatment of auditory hallucinations in schizophrenia. Eur Psychiatry J Assoc Eur Psychiatr.

[23] Hoy KE, Whitty D, Bailey N, Fitzgerald PB (2016). Preliminary investigation of the effects of γ-tACS on working memory in schizophrenia. J Neural Transm Vienna Austria.

[24] Haller N, Hasan A, Padberg F (2020). Gamma transcranial alternating current stimulation for treatment of negative symptoms in schizophrenia: Report of two cases. Asian J Psychiatry.

[25] Haller N, Hasan A, Padberg F (2020). Gamma transcranial alternating current stimulation in patients with negative symptoms in schizophrenia: a case series. Neurophysiol Clin Clin Neurophysiol.

[26] Sreeraj VS, Shanbhag V, Nawani H (2017). Feasibility of online neuromodulation using transcranial alternating current stimulation in schizophrenia. Indian J Psychol Med.

[27] Sreeraj VS, Shivakumar V, Sowmya S (2019). Online theta frequency transcranial alternating current stimulation for cognitive remediation in schizophrenia: a case report and review of literature. J ECT.

[28] Zhou D, Li A, Li X (2022). Effects of 40 Hz transcranial alternating current stimulation (tACS) on cognitive functions of patients with Alzheimer’s disease: a randomised, double-blind, sham-controlled clinical trial. J Neurol Neurosurg Psychiatry.

[29] Benussi A, Cantoni V, Grassi M (2022). Increasing brain gamma activity improves episodic memory and restores cholinergic dysfunction in Alzheimer’s disease. Ann Neurol.

[30] Mimura Y, Nishida H, Nakajima S (2021). Neurophysiological biomarkers using transcranial magnetic stimulation in Alzheimer’s disease and mild cognitive impairment: a systematic review and meta-analysis. Neurosci Biobehav Rev.

[31] Chaieb L, Antal A, Masurat F, Paulus W (2015). Neuroplastic effects of transcranial near-infrared stimulation (tNIRS) on the motor cortex. Front Behav Neurosci.

[32] Di Lazzaro V, Oliviero A, Tonali PA (2002). Noninvasive in vivo assessment of cholinergic cortical circuits in AD using transcranial magnetic stimulation. Neurology.

[33] Benussi A, Grassi M, Palluzzi F (2020). Classification accuracy of transcranial magnetic stimulation for the diagnosis of neurodegenerative dementias. Ann Neurol.

[34] Benussi A, Cantoni V, Cotelli MS (2021). Exposure to gamma tACS in Alzheimer’s disease: a randomized, double-blind, sham-controlled, crossover, pilot study. Brain Stimulat.

[35] Naro A, Corallo F, De Salvo S (2016). Promising role of neuromodulation in predicting the progression of mild cognitive impairment to dementia. J Alzheimers Dis JAD.

[36] Moussavi Z, Kimura K, Kehler L (2021). A novel program to improve cognitive function in individuals with dementia using transcranial alternating current stimulation (tACS) and tutored cognitive exercises. Front Aging.

[37] Sprugnoli G, Munsch F, Cappon D (2021). Impact of multisession 40Hz tACS on hippocampal perfusion in patients with Alzheimer’s disease. Alzheimers Res Ther.

[38] Bréchet L, Yu W, Biagi MC (2021). Patient-tailored, home-based non-invasive brain stimulation for memory deficits in dementia due to Alzheimer’s disease. Front Neurol.

[39] Liu Y, Tang C, Wei K (2022). Transcranial alternating current stimulation combined with sound stimulation improves the cognitive function of patients with Alzheimer’s disease: a case report and literature review. Front Neurol.

[40] Kim J, Kim H, Jeong H (2021). tACS as a promising therapeutic option for improving cognitive function in mild cognitive impairment: a direct comparison between tACS and tDCS. J Psychiatr Res.

[41] Palm U, Baumgartner C, Hoffmann L (2022). Single session gamma transcranial alternating stimulation does not modulate working memory in depressed patients and healthy controls. Neurophysiol Clin Clin Neurophysiol.

[42] Alexander ML, Alagapan S, Lugo CE (2019). Double-blind, randomized pilot clinical trial targeting alpha oscillations with transcranial alternating current stimulation (tACS) for the treatment of major depressive disorder (MDD). Transl Psychiatry.

[43] Haller N, Senner F, Brunoni AR (2020). Gamma transcranial alternating current stimulation improves mood and cognition in patients with major depression. J Psychiatr Res.

[44] Wilkening A, Kurzeck A, Dechantsreiter E (2019). Transcranial alternating current stimulation for the treatment of major depression during pregnancy. Psychiatry Res.

[45] Haller N, Senner F, Hasan A (2020). Gamma transcranial alternating current stimulation (γtACS) in obsessive-compulsive disorder: a case report. Fortschr Neurol Psychiatr.

[46] Kannen K, Aslan B, Boetzel C (2022). P300 modulation via transcranial alternating current stimulation in adult attention-deficit/hyperactivity disorder: a crossover study. Front Psychiatry.

[47] Dallmer-Zerbe I, Popp F, Lam AP (2020). Transcranial Alternating current stimulation (tACS) as a tool to modulate P300 amplitude in attention deficit hyperactivity disorder (ADHD): preliminary findings. Brain Topogr.

[48] Amouzadeh F, Sheikh M (2022). Impact of transcranial alternating current stimulation on working memory and selective attention in athletes with attention deficit hyperactivity disorder: randomized controlled trial. NeuroReport.

[49] Daughters SB, Yi JY, Phillips RD (2020). Alpha-tACS effect on inhibitory control and feasibility of administration in community outpatient substance use treatment. Drug Alcohol Depend.

[50] McKim TH, Dove SJ, Robinson DL (2021). Addiction history moderates the effect of prefrontal 10-Hz transcranial alternating current stimulation on habitual action selection. J Neurophysiol.

[51] Sabel BA, Zhou W, Huber F (2021). Non-invasive brain microcurrent stimulation therapy of long-COVID-19 reduces vascular dysregulation and improves visual and cognitive impairment. Restor Neurol Neurosci.

[52] Sterne JAC, Savović J, Page MJ (2019). RoB 2: a revised tool for assessing risk of bias in randomised trials. BMJ.

[53] Bora E, Yücel M, Pantelis C (2010). Cognitive impairment in schizophrenia and affective psychoses: implications for DSM-V criteria and beyond. Schizophr Bull.

[54] Gebreegziabhere Y, Habatmu K, Mihretu A (2022). Cognitive impairment in people with schizophrenia: an umbrella review. Eur Arch Psychiatry Clin Neurosci.

[55] Sheffield JM, Barch DM (2016). Cognition and resting-state functional connectivity in schizophrenia. Neurosci Biobehav Rev.

[56] Uhlhaas PJ, Singer W (2010). Abnormal neural oscillations and synchrony in schizophrenia. Nat Rev Neurosci.

[57] Chung DW, Geramita MA, Lewis DA (2022). Synaptic variability and cortical gamma oscillation power in schizophrenia. Am J Psychiatry.

[58] Shin Y-W, O’Donnell BF, Youn S, Kwon JS (2011). Gamma oscillation in schizophrenia. Psychiatry Investig.

[59] McCutcheon RA, Reis Marques T, Howes OD (2020). Schizophrenia-an overview. JAMA Psychiat.

[60] Konopaske GT, Lange N, Coyle JT, Benes FM (2014). Prefrontal cortical dendritic spine pathology in schizophrenia and bipolar disorder. JAMA Psychiat.

[61] Smucny J, Dienel SJ, Lewis DA, Carter CS (2022). Mechanisms underlying dorsolateral prefrontal cortex contributions to cognitive dysfunction in schizophrenia. Neuropsychopharmacol Off Publ Am Coll Neuropsychopharmacol.

[62] Lewis DA, Curley AA, Glausier JR, Volk DW (2012). Cortical parvalbumin interneurons and cognitive dysfunction in schizophrenia. Trends Neurosci.

[63] Dienel SJ, Schoonover KE, Lewis DA (2022). Cognitive dysfunction and prefrontal cortical circuit alterations in schizophrenia: developmental trajectories. Biol Psychiatry.

[64] Haenschel C, Bittner RA, Waltz J (2009). Cortical oscillatory activity is critical for working memory as revealed by deficits in early-onset schizophrenia. J Neurosci Off J Soc Neurosci.

[65] Howard MW, Rizzuto DS, Caplan JB (2003). Gamma oscillations correlate with working memory load in humans. Cereb Cortex N Y N.

[66] Jensen O, Kaiser J, Lachaux J-P (2007). Human gamma-frequency oscillations associated with attention and memory. Trends Neurosci.

[67] Chen C-MA, Stanford AD, Mao X (2014). GABA level, gamma oscillation, and working memory performance in schizophrenia. NeuroImage Clin.

[68] Dienel SJ, Lewis DA (2019). Alterations in cortical interneurons and cognitive function in schizophrenia. Neurobiol Dis.

[69] Christophel TB, Klink PC, Spitzer B (2017). The distributed nature of working memory. Trends Cogn Sci.

[70] Ratcliffe O, Shapiro K, Staresina BP (2022). Fronto-medial theta coordinates posterior maintenance of working memory content. Curr Biol CB.

[71] Miller EK, Lundqvist M, Bastos AM (2018). Working memory 2.0. Neuron.

[72] Roux F, Uhlhaas PJ (2014). Working memory and neural oscillations: alpha–gamma versus theta–gamma codes for distinct WM information?. Trends Cogn Sci.

[73] Hyafil A, Giraud A-L, Fontolan L, Gutkin B (2015). Neural cross-frequency coupling: connecting architectures, mechanisms, and functions. Trends Neurosci.

[74] Abubaker M, Al Qasem W, Kvašňák E (2021). Working memory and cross-frequency coupling of neuronal oscillations. Front Psychol.

[75] Lisman JE, Jensen O (2013). The θ-γ neural code. Neuron.

[76] Alekseichuk I, Turi Z, Amador de Lara G (2016). Spatial working memory in humans depends on theta and high gamma synchronization in the prefrontal cortex. Curr Biol CB.

[77] Barr MS, Rajji TK, Zomorrodi R (2017). Impaired theta-gamma coupling during working memory performance in schizophrenia. Schizophr Res.

[78] Lee TH, Kim M, Hwang WJ (2020). Relationship between resting-state theta phase-gamma amplitude coupling and neurocognitive functioning in patients with first-episode psychosis. Schizophr Res.

[79] APA (2013) Diagnostic and statistical manual of mental disorders. 5th edition. Washington, DC: American Psychiatric Association

[80] Atri A (2019). The Alzheimer’s disease clinical spectrum: diagnosis and management. Med Clin North Am.

[81] Kirova A-M, Bays RB, Lagalwar S (2015). Working memory and executive function decline across normal aging, mild cognitive impairment, and Alzheimer’s disease. BioMed Res Int.

[82] Jafari Z, Kolb BE, Mohajerani MH (2020). Neural oscillations and brain stimulation in Alzheimer’s disease. Prog Neurobiol.

[83] Başar E, Emek-Savaş DD, Güntekin B, Yener GG (2016). Delay of cognitive gamma responses in Alzheimer’s disease. NeuroImage Clin.

[84] Başar E, Femir B, Emek-Savaş DD (2017). Increased long distance event-related gamma band connectivity in Alzheimer’s disease. NeuroImage Clin.

[85] Goodman MS, Kumar S, Zomorrodi R (2018). Theta-gamma coupling and working memory in alzheimer’s dementia and mild cognitive impairment. Front Aging Neurosci.

[86] Yener G, Hünerli-Gündüz D, Yıldırım E (2022). Treatment effects on event-related EEG potentials and oscillations in Alzheimer’s disease. Int J Psychophysiol Off J Int Organ Psychophysiol.

[87] Casula EP, Pellicciari MC, Bonnì S (2022). Decreased frontal gamma activity in Alzheimer disease patients. Ann Neurol.

[88] Martorell AJ, Paulson AL, Suk H-J (2019). Multi-sensory gamma stimulation ameliorates Alzheimer’s-associated pathology and improves cognition. Cell.

[89] Iaccarino HF, Singer AC, Martorell AJ (2016). Gamma frequency entrainment attenuates amyloid load and modifies microglia. Nature.

[90] Dhaynaut M, Sprugnoli G, Cappon D (2022). Impact of 40 Hz transcranial alternating current stimulation on cerebral tau burden in patients with Alzheimer’s disease: a case series. J Alzheimers Dis JAD.

[91] Adaikkan C, Tsai L-H (2020). Gamma entrainment: impact on neurocircuits, glia, and therapeutic opportunities. Trends Neurosci.

[92] Gaubert S, Raimondo F, Houot M (2019). EEG evidence of compensatory mechanisms in preclinical Alzheimer’s disease. Brain J Neurol.

[93] Menardi A, Rossi S, Koch G (2022). Toward noninvasive brain stimulation 2.0 in Alzheimer’s disease. Ageing Res Rev.

[94] Pontecorvo MJ, Devous MD, Navitsky M (2017). Relationships between flortaucipir PET tau binding and amyloid burden, clinical diagnosis, age and cognition. Brain J Neurol.

[95] Kumar S, Zomorrodi R, Ghazala Z (2017). Extent of dorsolateral prefrontal cortex plasticity and its association with working memory in patients with Alzheimer disease. JAMA Psychiat.

[96] Jongsiriyanyong S, Limpawattana P (2018). Mild cognitive impairment in clinical practice: a review article. Am J Alzheimers Dis Other Demen.

[97] Petersen RC, Caracciolo B, Brayne C (2014). Mild cognitive impairment: a concept in evolution. J Intern Med.

[98] Fraga FJ, Mamani GQ, Johns E (2018). Early diagnosis of mild cognitive impairment and Alzheimer’s with event-related potentials and event-related desynchronization in N-back working memory tasks. Comput Methods Programs Biomed.

[99] Missonnier P, Deiber M-P, Gold G (2007). Working memory load-related electroencephalographic parameters can differentiate progressive from stable mild cognitive impairment. Neuroscience.

[100] Tülay EE, Güntekin B, Yener G (2020). Evoked and induced EEG oscillations to visual targets reveal a differential pattern of change along the spectrum of cognitive decline in Alzheimer’s disease. Int J Psychophysiol Off J Int Organ Psychophysiol.

[101] Otte C, Gold SM, Penninx BW (2016). Major depressive disorder. Nat Rev Dis Primer.

[102] Knight MJ, Baune BT (2018). Cognitive dysfunction in major depressive disorder. Curr Opin Psychiatry.

[103] Pan Z, Park C, Brietzke E (2019). Cognitive impairment in major depressive disorder. CNS Spectr.

[104] Strelets VB, Garakh ZV, Novototskii-Vlasov VY (2007). Comparative study of the gamma rhythm in normal conditions, during examination stress, and in patients with first depressive episode. Neurosci Behav Physiol.

[105] Maiella M, Casula EP, Borghi I (2022). Simultaneous transcranial electrical and magnetic stimulation boost gamma oscillations in the dorsolateral prefrontal cortex. Sci Rep.

[106] Koo PC, Berger C, Kronenberg G (2019). Combined cognitive, psychomotor and electrophysiological biomarkers in major depressive disorder. Eur Arch Psychiatry Clin Neurosci.

[107] Kaiser RH, Andrews-Hanna JR, Wager TD, Pizzagalli DA (2015). Large-scale network dysfunction in major depressive disorder: a meta-analysis of resting-state functional connectivity. JAMA Psychiat.

[108] Abramovitch A, Cooperman A (2015). The cognitive neuropsychology of obsessive-compulsive disorder: a critical review. J Obsessive-Compuls Relat Disord.

[109] Funch Uhre V, Melissa Larsen K, Marc Herz D (2022). Inhibitory control in obsessive compulsive disorder: a systematic review and activation likelihood estimation meta-analysis of functional magnetic resonance imaging studies. NeuroImage Clin.

[110] van Velzen LS, Vriend C, de Wit SJ, van den Heuvel OA (2014). Response inhibition and interference control in obsessive-compulsive spectrum disorders. Front Hum Neurosci.

[111] Park JY, Lee J, Park H-J (2012). Alpha amplitude and phase locking in obsessive-compulsive disorder during working memory. Int J Psychophysiol Off J Int Organ Psychophysiol.

[112] Ciesielski KT, Hämäläinen MS, Geller DA (2007). Dissociation between MEG alpha modulation and performance accuracy on visual working memory task in obsessive compulsive disorder. Hum Brain Mapp.

[113] Treu S, Gonzalez-Rosa JJ, Soto-Leon V (2021). A ventromedial prefrontal dysrhythmia in obsessive-compulsive disorder is attenuated by nucleus accumbens deep brain stimulation. Brain Stimul Basic Transl Clin Res Neuromodulation.

[114] Fuermaier ABM, Tucha L, Koerts J (2015). Cognitive impairment in adult ADHD–perspective matters!. Neuropsychology.

[115] Snyder SM, Hall JR (2006). A meta-analysis of quantitative EEG power associated with attention-deficit hyperactivity disorder. J Clin Neurophysiol Off Publ Am Electroencephalogr Soc.

[116] Arns M, Conners CK, Kraemer HC (2013). A decade of EEG theta/beta ratio research in ADHD: a meta-analysis. J Atten Disord.

[117] Saad JF, Kohn MR, Clarke S (2018). Is the theta/beta eeg marker for ADHD inherently flawed?. J Atten Disord.

[118] Kiiski H, Bennett M, Rueda-Delgado LM (2020). EEG spectral power, but not theta/beta ratio, is a neuromarker for adult ADHD. Eur J Neurosci.

[119] Picken C, Clarke AR, Barry RJ (2020). The theta/beta ratio as an index of cognitive processing in adults with the combined type of attention deficit hyperactivity disorder. Clin EEG Neurosci.

[120] Deiber M-P, Hasler R, Colin J (2020). Linking alpha oscillations, attention and inhibitory control in adult ADHD with EEG neurofeedback. NeuroImage Clin.

[121] Sari Gokten E, Tulay EE, Beser B (2019). Predictive value of slow and fast EEG oscillations for methylphenidate response in ADHD. Clin EEG Neurosci.

[122] Hasler R, Perroud N, Meziane HB (2016). Attention-related EEG markers in adult ADHD. Neuropsychologia.

[123] Kaiser A, Aggensteiner P-M, Baumeister S (2020). Earlier versus later cognitive event-related potentials (ERPs) in attention-deficit/hyperactivity disorder (ADHD): a meta-analysis. Neurosci Biobehav Rev.

[124] Andrew C, Fein G (2010). Event-related oscillations versus event-related potentials in a P300 task as biomarkers for alcoholism. Alcohol Clin Exp Res.

[125] Popp F, Dallmer-Zerbe I, Philipsen A, Herrmann CS (2019). Challenges of P300 modulation using transcranial alternating current stimulation (tACS). Front Psychol.

[126] Hwang K, Ghuman AS, Manoach DS (2014). Cortical neurodynamics of inhibitory control. J Neurosci Off J Soc Neurosci.

[127] Passarotti AM, Sweeney JA, Pavuluri MN (2010). Neural correlates of response inhibition in pediatric bipolar disorder and attention deficit hyperactivity disorder. Psychiatry Res.

[128] Bodkyn CN, Holroyd CB (2019). Neural mechanisms of affective instability and cognitive control in substance use. Int J Psychophysiol Off J Int Organ Psychophysiol.

[129] Kim-Spoon J, Deater-Deckard K, Brieant A (2019). Brains of a feather flocking together? Peer and individual neurobehavioral risks for substance use across adolescence. Dev Psychopathol.

[130] Kim-Spoon J, Herd T, Brieant A (2021). Bidirectional links between adolescent brain function and substance use moderated by cognitive control. J Child Psychol Psychiatry.

[131] Billieux J, Gay P, Rochat L (2010). Lack of inhibitory control predicts cigarette smoking dependence: evidence from a non-deprived sample of light to moderate smokers. Drug Alcohol Depend.

[132] Li CR, Luo X, Yan P (2009). Altered impulse control in alcohol dependence: neural measures of stop signal performance. Alcohol Clin Exp Res.

[133] Luijten M, Machielsen MWJ, Veltman DJ (2014). Systematic review of ERP and fMRI studies investigating inhibitory control and error processing in people with substance dependence and behavioural addictions. J Psychiatry Neurosci JPN.

[134] Bel-Bahar TS, Khan AA, Shaik RB, Parvaz MA (2022). A scoping review of electroencephalographic (EEG) markers for tracking neurophysiological changes and predicting outcomes in substance use disorder treatment. Front Hum Neurosci.

[135] Campanella S, Pogarell O, Boutros N (2014). Event-related potentials in substance use disorders: a narrative review based on articles from 1984 to 2012. Clin EEG Neurosci.

[136] Ceballos NA, Bauer LO, Houston RJ (2009). Recent EEG and ERP findings in substance abusers. Clin EEG Neurosci.

[137] Premraj L, Kannapadi NV, Briggs J (2022). Mid and long-term neurological and neuropsychiatric manifestations of post-COVID-19 syndrome: a meta-analysis. J Neurol Sci.

[138] Ceban F, Ling S, Lui LMW (2022). Fatigue and cognitive impairment in Post-COVID-19 Syndrome: a systematic review and meta-analysis. Brain Behav Immun.

[139] Crivelli L, Palmer K, Calandri I (2022). Changes in cognitive functioning after COVID-19: a systematic review and meta-analysis. Alzheimers Dement J Alzheimers Assoc.

[140] Hameed S, Saleem S, Sajjad A (2022). Spectrum of EEG abnormalities in COVID-19 patients. J Clin Neurophysiol Off Publ Am Electroencephalogr Soc.

[141] Kubota T, Gajera PK, Kuroda N (2021). Meta-analysis of EEG findings in patients with COVID-19. Epilepsy Behav EB.

[142] Chang C-H, Chen S-J, Chen Y-C, Tsai H-C (2023). A 30-year-old woman with an 8-week history of anxiety, depression, insomnia, and mild cognitive impairment following COVID-19 who responded to accelerated bilateral theta-burst transcranial magnetic stimulation over the prefrontal cortex. Am J Case Rep.

[143] Noda Y, Sato A, Fujii K (2023). A pilot study of the effect of transcranial magnetic stimulation treatment on cognitive dysfunction associated with post COVID-19 condition. Psychiatry Clin Neurosci.

[144] Frohlich F, Townsend L (2021). Closed-loop transcranial alternating current stimulation: towards personalized non-invasive brain stimulation for the treatment of psychiatric illnesses. Curr Behav Neurosci Rep.

[145] Klírová M, Voráčková V, Horáček J (2021). Modulating inhibitory control processes using individualized high definition theta transcranial alternating current stimulation (HD θ-tACS) of the anterior cingulate and medial prefrontal cortex. Front Syst Neurosci.

[146] Ketz N, Jones AP, Bryant NB (2018). Closed-loop slow-wave tACS improves sleep-dependent long-term memory generalization by modulating endogenous oscillations. J Neurosci Off J Soc Neurosci.

[147] Stecher HI, Notbohm A, Kasten FH, Herrmann CS (2021). A comparison of closed loop vs. fixed frequency tACS on modulating brain oscillations and visual detection. Front Hum Neurosci.

[148] Bahar-Fuchs A, Martyr A, Goh AM (2019). Cognitive training for people with mild to moderate dementia. Cochrane Database Syst Rev.

[149] Bellani M, Ricciardi C, Rossetti MG (2019). Cognitive remediation in schizophrenia: the earlier the better?. Epidemiol Psychiatr Sci.

[150] Hill NTM, Mowszowski L, Naismith SL (2017). Computerized cognitive training in older adults with mild cognitive impairment or dementia: a systematic review and meta-analysis. Am J Psychiatry.

[151] Janssens SEW, Sack AT (2021). Spontaneous fluctuations in oscillatory brain state cause differences in transcranial magnetic stimulation effects within and between individuals. Front Hum Neurosci.

[152] Jones KT, Johnson EL, Tauxe ZS, Rojas DC (2020). Modulation of auditory gamma-band responses using transcranial electrical stimulation. J Neurophysiol.

[153] Jones KT, Johnson EL, Gazzaley A, Zanto TP (2022). Structural and functional network mechanisms of rescuing cognitive control in aging. Neuroimage.

[154] Chai Y, Sheng J, Bandettini PA, Gao J-H (2018). Frequency-dependent tACS modulation of BOLD signal during rhythmic visual stimulation. Hum Brain Mapp.

[155] Abellaneda-Pérez K, Vaqué-Alcázar L, Perellón-Alfonso R (2019). Differential tDCS and tACS effects on working memory-related neural activity and resting-state connectivity. Front Neurosci.

[156] Turi Z, Mittner M, Lehr A (2020). θ-γ cross-frequency transcranial alternating current stimulation over the trough impairs cognitive control. eNeuro.

[157] Yakubov B, Das S, Zomorrodi R (2022). Cross-frequency coupling in psychiatric disorders: a systematic review. Neurosci Biobehav Rev.

